# G Protein-Coupled Receptor Signaling Analysis Using Homogenous Time-Resolved Förster Resonance Energy Transfer (HTRF^®^) Technology

**DOI:** 10.3390/ijms15022554

**Published:** 2014-02-13

**Authors:** Lenea Nørskov-Lauritsen, Alex Rojas Bie Thomsen, Hans Bräuner-Osborne

**Affiliations:** Department of Drug Design and Pharmacology, Faculty of Health and Medical Sciences, University of Copenhagen, Fruebjergvej 3, Mailbox 10, Copenhagen DK-2100, Denmark; E-Mails: lnl@sund.ku.dk (L.N.-L.); alex.molpharm@gmail.com (A.R.B.T.)

**Keywords:** G protein-coupled receptor (GPCR), signaling pathway, high throughput screening (HTS), homogenous time-resolved Förster resonance energy transfer (HTRF^®^), d-myo-inositol 1-phosphate (IP_1_), cyclic adenosine 3′,5′-monophosphate (cAMP), extracellular signal-regulated kinases (ERK1/2)

## Abstract

Studying multidimensional signaling of G protein-coupled receptors (GPCRs) in search of new and better treatments requires flexible, reliable and sensitive assays in high throughput screening (HTS) formats. Today, more than half of the detection techniques used in HTS are based on fluorescence, because of the high sensitivity and rich signal, but quenching, optical interferences and light scattering are serious drawbacks. In the 1990s the HTRF^®^ (Cisbio Bioassays, Codolet, France) technology based on Förster resonance energy transfer (FRET) in a time-resolved homogeneous format was developed. This improved technology diminished the traditional drawbacks. The optimized protocol described here based on HTRF^®^ technology was used to study the activation and signaling pathways of the calcium-sensing receptor, CaSR, a GPCR responsible for maintaining calcium homeostasis. Stimulation of the CaSR by agonists activated several pathways, which were detected by measuring accumulation of the second messengers d-myo-inositol 1-phosphate (IP_1_) and cyclic adenosine 3′,5′-monophosphate (cAMP), and by measuring the phosphorylation of extracellular signal-regulated kinase 1 and 2 (ERK1/2). Here we show how an optimized HTRF^®^ platform with numerous advantages compared to previous assays provides a substantial and robust mode of investigating GPCR signaling. It is furthermore discussed how these assays can be optimized and miniaturized to meet HTS requirements and for screening compound libraries.

## 1. Introduction

G protein-coupled receptor (GPCR) signaling is multidimensional and can act through activation of G proteins as well as other intracellular proteins such as β-arrestins [1]. In recent years several lines of evidence suggest that ligands can stabilize different conformations of the same receptor leading to a differential activation pattern called biased receptor signaling [2]. This new concept of signaling bias allowing one ligand to act differentially on multiple signaling pathways has increased the requirements of screening assays. Furthermore, the increased focus on allosteric compounds that modulate the affinity and/or efficacy of the orthosteric ligand and the search for endogenous ligands (deorphanization) of GPCRs increases the requirement for more rigorous high throughput screening (HTS) platforms. Screening assays therefore have to be highly reliable and robust, yet flexible, to enable identification of new drugs and drug targets. In an industrial setting, HTS formats are desirable because of the increasing size of compound screening libraries and number of targets [3]. In academia the demands of screening assays have also increased due to large drug discovery programs such as the NIH Molecular Libraries Initiative [4]. In both settings, what is required are flexible, sensitive, and cost-effective functional cell-based assays that have high throughput and fewer false-negative or false-positive results [5]. Assays based on label-free technology that can detect activation of several pathways at the same time are under development [6] but are not fully optimized and validated for HTS formats [1]. Furthermore, the high running cost currently delays its implementation into the mainstream [7]. Finally, label-free assays will not directly delineate the signaling pathway(s) activated by the ligand which subsequently have to be determined e.g., by use of pathway inhibitors [8].

Traditional binding and uptake assays using radioligands are becoming less common because of an environmental burden, hazards to human health, limits in detecting allosteric modulation, and heterogeneous setups with several washing steps [1]. Instead functional assays based on enzymatic, luminescence or fluorescence methods have been developed [9]. These methods have been optimized to be homogenous (non-separation techniques). Homogeneity allows a high level of automation as well as the study of low affinity interactions which makes these methods suitable for HTS [9]. Fluorescence-based techniques remain very popular in HTS settings because of a combination of the high amplitude of the fluorescent signal (based on quantum yield, energy, lifetime, and anisotropy) and the high sensitivity [10]. However, there are several drawbacks to fluorescence methods including quenching, optical interferences, fluorescence of screening compounds or media, and light scattering [9,11] leading to low sensitivity and low signal-to-background ratios. Thereby the amount of false-positive or false-negative hits is increased. High sensitivity is especially important when studying GPCR targets that are poorly expressed and/or do not couple strongly to endogenous G proteins or other signaling molecules. Much research in improving fluorescence technologies has been conducted and in the 1990s the HTRF^®^ technology based on Förster resonance energy transfer (FRET) in a time-resolved format was marketed [12].

Energy migration between molecules was already discussed in the 1920s. In 1946 Theodor Förster published his calculations proving that energy between two fluorescent molecules with overlapping spectra can be transferred due to a resonance effect [13]. This means that excitation of a donor molecule can transfer energy to an acceptor molecule in close proximity, which then emits light at a different wavelength than the donor. However, for this to occur there are three requirements: (1) an overlap between donor emission and acceptor excitation spectra and (2) the dipole orientations of the two fluorophores should be compatible. The dipoles describe the oscillating fields surrounding the fluorophores upon excitation and the coupling is a function of the relative orientation of both, meaning that two perpendicular fields will cancel one another [14]. The orientation of the dipoles can be described by the orientation factor, κ^2^, which is directly proportional to the energy transfer efficiency [15]; (3) Lastly, the two fluorophores should be located in close proximity with each other [14]. The distance between the fluorophores leading to 50% energy transfer is called *R*_0_ and the FRET efficiency is strongly dependent on this value. Fluorophores located at half the *R*_0_ distance give rise to 98.5% energy transfer whereas a double *R*_0_ distance gives rise to only 1.5% energy transfer [15]. Some HTRF^®^ pairs allow measuring of FRET over long distances with *R*_0_ values of up to 100 Å [16]. The *R*_0_ can be calculated for the donor-acceptor pair. HTRF^®^ is based on the basic FRET principle but have been optimized to circumvent or decrease the importance of the traditional FRET requirements.

**Figure 1.**
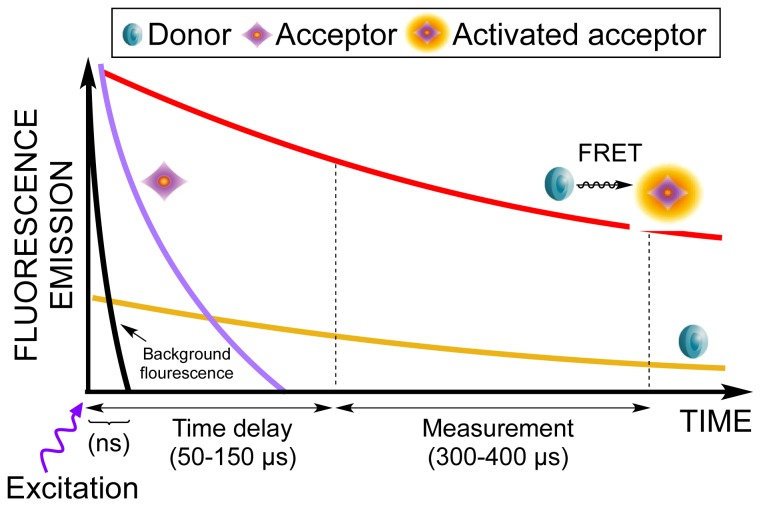
Principle of time-resolved detection. After an initial excitation pulse, a delay of 50–150 μs is introduced. Background fluorescence from, e.g., media, ligands and/or buffer will fade within nanoseconds (ns). When starting the measurement after the time delay only the long emitting donor fluorophore and the activated acceptor will emit light.

HTRF^®^ is a time-resolved measurement meaning that a time delay between excitation of donor and readout of acceptor-emission has been introduced. Thereby autofluorescence from, e.g., media, ligands and/or buffers is avoided (Figure 1). This is only possible because of a long emitting donor molecule.

Other time-resolved techniques include quenching resonance energy transfer, QRET™ (BN Products&Services, Turku, Finland) as well as LANCE^®^ (PerkinElmer, Waltham, MA, USA) and LanthaScreen™ (Life Technologies Europe, Nærum, Denmark) based on time-resolved FRET (TR-FRET). QRET™ is a single label approach using a donor molecule and a soluble quencher, which can quench unbound donor [17,18]. LANCE^®^ is based on an europium chelate and two different acceptor molecules of which *U*Light™ (PerkinElmer, Waltham, MA, USA) is the second generation which has the advantage of a molecular weight below 800 Da [19]. LanthaScreen™ technology used to be based on europium as donor and allophycocyanin as acceptor, but has been optimized to use terbium as donor and the smaller fluorophore fluorescein as acceptor [20].

Europium was the first long emitting donor applied [21] and as a second generation, terbium was developed. Both have extremely long half-lives (300 μs–1 ms) compared to other fluorophores [12]. They are compatible with the same acceptor molecules, but terbium is 10–20 times brighter than europium giving rise to higher sensitivity [5]. Europium and terbium are both rare earth metals belonging to the lanthanide family that themselves have poor absorption coefficients [16]. In order to attach the rare earth metals to e.g., an antibody, it is necessary to use chemical complexes such as chelates or cryptates. These complexes “capture” the ions. In addition, chelation or cryptate caging improves the fluorescent properties of the rare earth metals [21,22]. Chelates such as polycarboxylate complexes have been shown to tightly bind both terbium and europium [23]. The so-called cryptate macromolecule constitutes a three-dimensional cage composed of pyridine rings [12]. Photobleaching of these cryptated fluorophores is not easy, but can occur after 2 min which however is a much larger time span than the one used in HTRF^®^ measurements (Figure 1) [24]. They also have an increased stability and function as antennas that absorb light from all directions thereby avoiding the obligatory alignment of dipole-dipole orientations, as is required in traditional FRET [12,24,25]. Chelates are not as stable as cryptates in acidic environments and are prone to exchange the positively charged lanthanide with ions like Mg^2+^ [26].

The first acceptor used for HTRF^®^ was allophycocyanin or XL665, a 105 kDa phycobilliprotein pigment derived from red algae [27]. Since then, a second-generation acceptor, d2, has been developed. The d2 is an organic dye with the same photophysical properties as XL665, but 100-times smaller in size, avoiding possible steric hindrance issues with XL665 [28]. In addition, the d2 acceptor has significantly increased the stability of the HTRF^®^ assays. Both acceptors emit light at 665 nm thereby avoiding interference with most proteins and compounds that usually emit below 500 nm [28].

The output of the HTRF^®^ assays are detected in a ratiometric manner, where the emitted light from the acceptor is divided by the emitted light from the donor (FRET ratio = acceptor emission/donor emission). This output corrects for variability from well-to-well and media composition variations (e.g., when screening compound libraries or testing different cell densities) as well as quenching from other assay components [5].

To study the activation and signaling pathways of GPCRs sensitive, reliable, and fast assays with a high dynamic range are wanted. G_q_ coupled receptors generate the second messenger d-myo-inositol 1,4,5-trisphosphate (IP_3_) upon ligand stimulation through the enzyme phospholipase C. IP_3_ triggers calcium release within the cell and is rapidly metabolized into d-myo-inositol bisphosphate (IP_2_), then d-myo-inositol 1-phosphate (IP_1_) and eventually d-myo-inositol. Traditional assays detecting intracellular calcium release or using radioactive labeled d-myo-inositol have been used to study G_q_ coupled receptors. However, most are not applicable in a HTS format [11]. Intracellular calcium measurement using e.g., a Fluorescent Imaging Plate Reader (FLIPR^®^, Molecular Devices, Sunnyvale, CA, USA) has been applied in HTS format [29], but is based on fluorescence measurements that share the same drawbacks (e.g., background fluorescence and quenching) as traditional FRET methods. Furthermore, these calcium assays do not allow screening of inverse agonists or slow-binding ligands [30] and are not specific for G_q_ activity since other mechanisms can lead to increases in intracellular calcium. Scintillation proximity assays (SPA) using beads and radioactive labeled d-myo-inositol have been applied in high-throughput measurement of GPCR activity [31], but still have the drawbacks of using hazardous materials and a heterogeneous setup.

**Figure 2.**
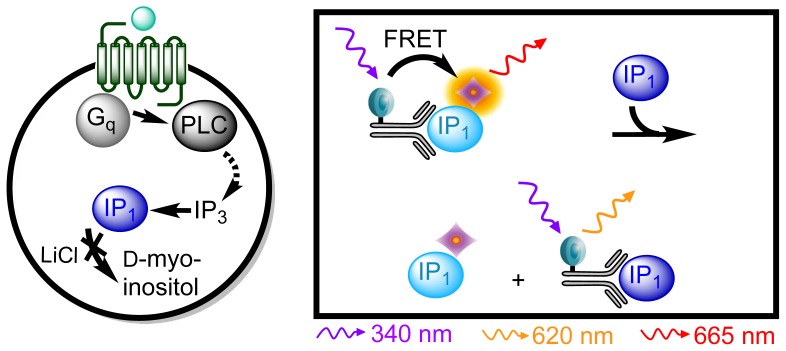
IP-One assay principle. (**Left**) Upon ligand stimulation of the GPCR activation of the G_q_ protein occurs through activation of phospholipase C (PLC) leading to the formation of inositol 1,4,5-trisphosphate (IP_3_). IP_3_ is degraded into IP_1_ and then d-myo-inositol in a rapid manner. LiCl inhibits this last step in the degradation; (**Right**) The endogenous IP_1_ competes with acceptor-labeled IP_1_ for the binding sites of a donor-labeled IP_1_ antibody. Hence accumulated IP_1_ will lead to a decrease in FRET signal.

An assay based on HTRF^®^ technology using lithium chloride (LiCl), which inhibits IP_1_-degradation [32], and a fluorophore labeled IP_1_ specific antibody has proven effective in studying G_q_ coupled receptor activation and constitutive activity [11]. This IP-One assay (Cisbio Bioassays, Codolet, France) measures the accumulation of IP_1_ instead of the transient second messengers IP_3_ and IP_2_ and is proven effective as a surrogate measure of GPCR activation [11]. The detection is based on competition between acceptor-labeled IP_1_ and IP_1_ produced by the cell [33] (Figure 2).

**Figure 3.**
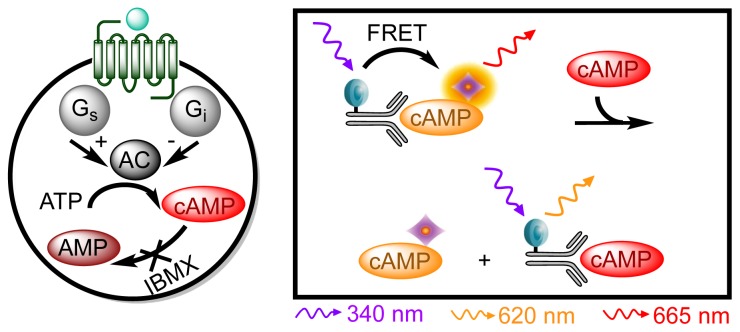
cAMP dynamic 2 assay principle. (**Left**) Upon ligand stimulation of the GPCR activation of G_s_ or G_i_ can occur. The G proteins either stimulate (G_s_) or inhibit (G_i_) adenylate cyclase (AC), which converts ATP into cAMP. Degradation of cAMP is inhibited by IBMX; (**Right**) Endogenous cAMP competes with acceptor-labeled cAMP for the binding sites of a donor-labeled cAMP antibody. Hence accumulated cAMP will lead to a decrease in FRET signal and an inhibition of cAMP will increase the FRET signal.

G_i_ and G_s_ coupled receptors modulate adenlylate cyclase (AC) activity, which is responsible for the formation of the second messenger cyclic adenosine 3′,5′-monophosphate (cAMP) from adenosine triphosphate (ATP). G_i_ activity inhibits AC thereby decreasing cAMP concentration whereas G_s_ increases the concentration by stimulating AC. The same detection principle as above has been applied for a cAMP assay. This cAMP dynamic 2 kit (Cisbio Bioassays, Codolet, France) is applicable for studying both G_i_ and G_s_ coupled GPCRs [34]. Here, 3-isobutyl-1-methylxanthine (IBMX) is responsible for cAMP accumulation by inhibiting phosphodiesterase driven degradation to 5′-AMP (Figure 3). When studying G_i_ activation, forskolin, which directly stimulates cAMP formation, is used to generate a basal level of second messenger that in turn can be inhibited by activation of a G_i_ coupled GPCR. The HTRF^®^ based cAMP assays can be used in a HTS assay format and show many advantages including few pipetting steps, broad linear range, high signal-to-noise ratio, and the additional advantage of the previously described ratiometric detection [34].

**Figure 4.**
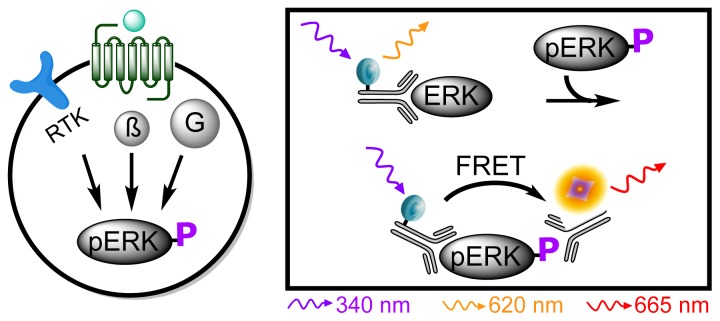
Phospho-ERK assay principle. (**Left**) Upon ligand stimulation of the GPCR various pathways can be activated and lead to the phosphorylation of ERK1/2. Activation can occur by involvement of G proteins (G), via an interaction with β-arrestins (β) or transactivation of receptor tyrosine kinases (RTK); (**Right**) A donor-labeled antibody binds ERK1/2 whereas an acceptor-labeled antibody binds phosphorylated ERK1/2 (pERK) giving rise to a FRET signal.

Another important factor involved in GPCR signaling is activation of protein kinase cascades leading to the phosphorylation of extracellular signal-regulated kinase 1 and 2 (ERK1/2). The phosphorylation can be triggered by several pathways including G protein activation, β-arrestin activation or by transactivation of receptor tyrosine kinases (RTKs) [35,36]. Traditionally, these phosphorylated proteins have been assessed using Western blotting that requires very time-consuming steps [37]. An HTRF^®^ based assay, Phospho-ERK (Cisbio Bioassays, Codolet, France) has been developed for measuring the level of phosphorylation of ERK1/2. This sandwich assay uses two antibodies; one labeled with a donor fluorophore targeting ERK1/2 and another antibody labeled with an acceptor fluorophore targeting the phosphorylated and activated kinases (pERK1/2). Hence phosphorylation of ERK1/2 will give rise to a FRET signal [38] (Figure 4).

This HTRF^®^ platform thus allows measurement of the major GPCR signaling pathways in a highly efficient and affordable HTS format. The time-resolved detection provides numerous advantages compared to previous fluorescence-based assays and enable both detailed studies of GPCR signaling and screening of large compound libraries.

Several signaling pathways activated by GPCRs are being investigated by our group using assays based on the HTRF^®^ technology. As a model, we here describe how these assays have been successfully employed and optimized to study the signaling of the calcium-sensing receptor (CaSR). Detailed and optimized protocols with useful comments are given for all assays allowing readers to easily apply these methods.

## 2. Results and Discussion

**Figure 5.**
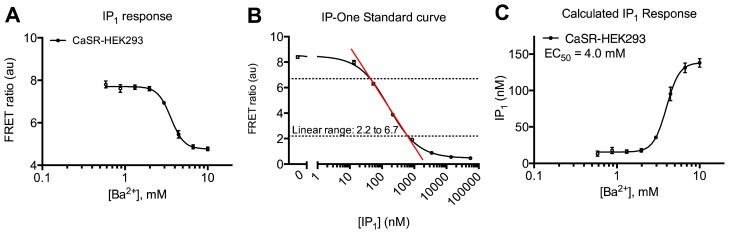
Characterization of barium-induced G_q_ pathway activation in CaSR-HEK293 cells using HTRF^®^ based assay. (**A**) Concentration-response curve of barium in the IP-One assay using 10^4^ cells/well and stimulation for 1 h at 37 °C. Decrease in FRET signal is inversely proportional to IP_1_ accumulation; (**B**) IP-One standard curve showing concentration-response curve of the IP-One Calibrator diluted in a 1:1 mixture of Ligand and Assay buffer; (**C**) Concentration-response curve of barium from (**A**) showing the amount of accumulated IP_1_ calculated by use of the standard curve in (**B**). Data are mean ± S.D. of triplicate determinations and representative for three independent experiments.

**Figure 6.**
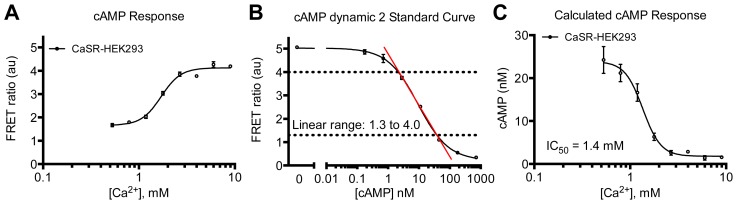
Characterization of calcium-induced G_i_ pathway activation in CaSR-HEK293 cells using HTRF^®^ based assay. (**A**) Concentration-response curve of calcium in the presence of 5 μM forskolin using 2 × 10^4^ cells/well and stimulation for 30 min at RT shown as FRET ratio. Increase in FRET signal is proportional to inhibition of cAMP production; (**B**) cAMP dynamic 2 standard curve showing concentration response curve of the cAMP standard diluted in a 1:1 mixture of Assay and Ligand buffer containing 1 μM IBMX and 10 μM forskolin; (**C**) Concentration-response curve of calcium in the presence of 5 μM forskolin shown as cAMP concentration. Data shown are calculated from the data in **Panel A** by the use of the standard curve in **Panel B**. Data are mean ± S.D. of triplicate determinations and representative for three independent experiments.

**Figure 7.**
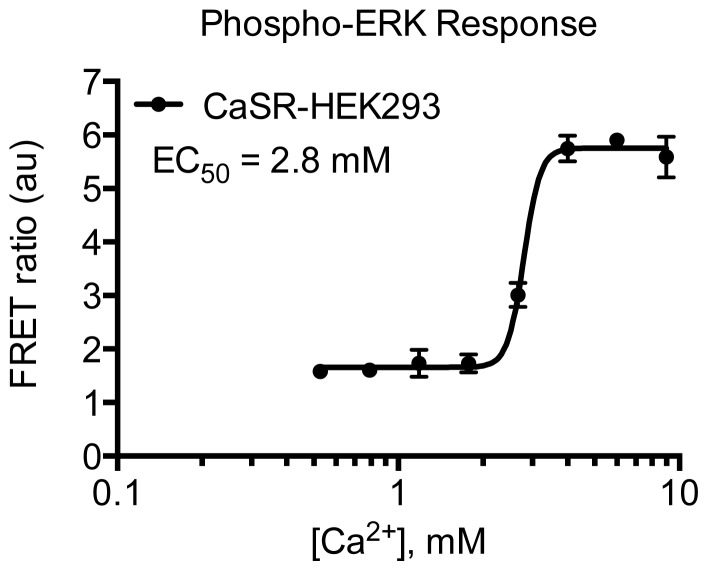
Characterization of calcium-induced phosphorylation of ERK1/2 in CaSR-HEK293 cells using HTRF^®^ based assay. Concentration-response curve of calcium in the Phospho-ERK1/2 HTRF^®^ assay using 5 × 10^4^ cells/well (plated 24 h before) and 15 min stimulation. Increase in FRET signal is proportional to the phosphorylation of ERK1/2. Data are mean ± S.D. of triplicate determinations and representative for three independent experiments.

CaSR is a class C GPCR involved in physiological calcium homeostasis and an interesting drug target in parathyroid hormone related diseases [39]. Here the HTRF^®^ technology has been applied to study the receptor’s multidimensional signaling properties. It is shown that upon agonist stimulation with either calcium or barium that CaSR-HEK293 cells induce both activation of the G_q_ (IP_1_ accumulation) (Figure 5) and G_i_ (inhibition of cAMP production) (Figure 6) pathways. The potency of barium for IP_1_ production was 4.0 mM and has previously been determined to be 3 or 6.2 mM in AtT-20 cells (derived from a mouse pituitary tumor) or bovine parathyroid cells, respectively [40,41]. In the same study, the potency of calcium to inhibit cAMP generation was found to be 1.9 mM that correlates well with our result of 1.4 mM [40]. Stimulation with calcium also leads to phosphorylation of ERK1/2 (Figure 7) with a potency of 2.8 mM. No significant effect was seen on non-transfected cells in any of the assays (data not shown). The applied optimized protocols are thoroughly explained in the Experimental Section.

In our study, a stably transfected cell line with high expression level has been chosen as a model system. However, thyroid cells with endogenous CaSR expression have been used to investigate the signaling pathways of different ligands for this receptor [42,43] making it possible with this optimized technology to study receptors in their endogenous environment.

**Figure 8.**
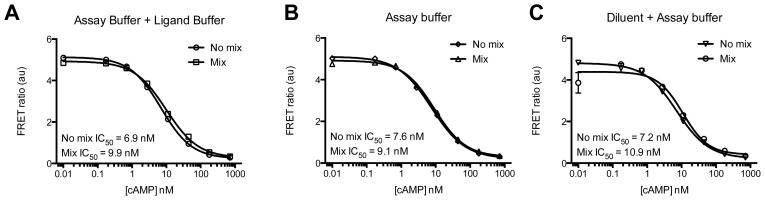
Standard curve of cAMP in different buffers with or without mixing of fluorophores for detection. cAMP Standard was diluted in (**A**) a 1:1 ratio of Assay buffer (HBSS + 20 mM HEPES pH 7.4) and Ligand Buffer (HBSS + 20 mM HEPES pH 7.4 + 1 μM IBMX + 10 μM forskolin; (**B**) Assay Buffer; or (**C**) Diluent (from kit) and Assay Buffer. Fluorophores were then added either as a pre-mixture or one at a time. Data are mean ± S.D. of triplicate determinations.

During assay optimization it was found that there was no difference between pre-mixing or not mixing the fluorophores and the buffer composition for the cAMP assay (Figure 8). For the determination of IP_1_ pre-mixing of fluorophores did not affect the results, but choice of buffer shifted the basal FRET ratio values (data not shown).

The conditions of each assay have been carefully optimized with regards to cell density, stimulation time and temperature as well as buffer composition (see Table 1) to achieve the best read-out with regards to dynamic range and signal-to-background ratio. It is evident that similar conditions would be preferable for comparing the different signaling pathways. However, due to e.g., the need of obtaining IP_1_ and cAMP levels within the linear range (Figures 5B and 6B) and of specific enzyme activators/inhibitors in the two assays, it is not possible to make all parameters identical. Nevertheless, the same pH calibrated buffer has been used in all assays with the addition of different enzyme activators/inhibitors (Table 1). This is particularly important for the CaSR as it has been shown to be modulated by extracellular pH, sodium concentration and ionic strength [44,45]. The different assay conditions and sensitivities might lead to “observational bias” that should not be misinterpreted with “agonist bias” [46]. This problem is not restricted to the HTRF^®^ based assays presented here, but is inherent to basically all types of signaling pathway assays, which almost always will differ in their experimental setup. The best way to distinguish between “observational bias” and “agonist bias” is to include one or more standard agonist(s) (e.g., endogenous agonist(s)) and then compare pharmacological profiles of novel agonists with this standard using bias plots [46].

The HTRF^®^ technology provides many advantages compared to traditional FRET based methods that are subject to limitations caused by quenching, fluorescence of library compounds and optical interference like inner filter effects or light diffusion by particles [10]. In HTRF^®^ technology most of these limitations have been circumvented by optimizing donor/acceptor properties, both chemical and spectral, and introducing time-resolved measurement. The ratiometric detection method has been established to correct for well-to-well and media variation as well as inner filter effects of the donor fluorophore. Nevertheless, a possible inner filter effect at the acceptor emission wavelength cannot be corrected by this method. It is however argued that only a small amount of library compounds will absorb light at this high wavelength (665 nm) and hence this effect is less probable [10]. The sensitivity of the HTRF^®^ technology is much higher compared to traditional methods exemplified by a detectable G_q_ response of the closely related GPRC6A receptor when using HTRF technology compared to no response in the more traditional fluorescence-based Fluo-4 NW calcium assay [47].

It is clear that these homogenous format assays are less time-consuming compared to traditional assays with washing steps. It is of great importance that the data achieved are within the linear range of the standard curve, which requires rigorous optimization of parameters such as: the number of cells per well, stimulation time, and/or concentration of degradation inhibitors (IMBX or LiCl), and/or the cAMP stimulator forskolin. For instance, we have observed relatively big differences in forskolin concentration to be used when testing for cAMP inhibition (G_i_ activation) depending on cell line and the GPCR assayed. This should be taken into account when setting up these assays, like in every other assay. In case of the IP-One assay, it has been optimized and miniaturized to a one-day HTS format using 1536-well plates, cryopreserved cells and a dispense-only format with good correlation of compound properties with traditional calcium or [^3^H]-IP_3_ measurements [33,48,49]. As evident from the protocols detailed above, the HTRF^®^ assays of IP_1_ and cAMP are homogeneous assays with true HTS capacity. In contrast the ERK1/2 assay described here requires ligand addition to adherent cells in 96-well plates followed by cell lysis and transfer of lysate to a 384-well plate where the HTRF^®^ reagents are added. This ERK1/2 assay thus have lower throughput than the IP_1_ and cAMP assays, but still have much higher throughput than the Western blot assays commonly used and can be miniaturized to a one-plate assay protocol according to the manufacturer [38].

The HTRF^®^ cAMP assay is advantageous because of its short incubation time. It is also a better alternative to radioactive assays with hazardous waste and reporter gene assays, where transcription activation can be influenced by several compound interactions [34]. Another study has showed that this assay can be used to eliminate false-positive results and has better coefficient of variation (CV) and statistical performance (*Z*′ factor). However, it is noted that agonists and antagonists cannot be screened at the same time since the cells are lysed in the three described assays [50]. Further, lysis of the cells makes it impossible to do real-time kinetic studies, so it should be considered if end-point measurements are sufficient in the relevant case. The same authors were concerned that the HTRF^®^ cAMP assay would not be sensitive enough to study cell lines expressing receptors in a low level like endogenous receptors [50]. Nevertheless biased signaling of the CaSR has been studied in cells endogenously expressing the receptor in low levels using the cAMP assay [43].

When studying (biased) signaling of GPCR ligands an HTRF^®^ platform could be beneficial since it is easy to use all three assays where workflow and readout are similar. At present there are no other technologies (e.g., QRET, LANCE^®^ or LanthaScreen™) providing assays for both IP_1_, cAMP and pERK1/2 measurements [17,19,20]. Applying a method like label-free detection would require an extensive amount of optimization using different pathway inhibitors and would be complex for a target with many biased ligands [8].

Mixing of the fluorophores before addition to the plate reduces duration of assay and chance of pipetting error, but could affect antibody equilibrium if shorter incubation times are applied. We recommended testing both with and without mixing for the first time in the relevant protocol.

Finally it should be noted that since all these assays are antibody-based it makes them highly dependent on specific antibodies. If the antibody affinity for labeled *versus* non-labeled second messenger is different or if the phospho-specific antibody is not specific enough this will lead to biased results [34,51]. Furthermore, the quality of the antibodies and fluorescent-labeled signaling molecules can vary from batch to batch and it is thus important to generate standard curves for each batch.

## 3. Experimental Section

### 3.1. Materials

- IP-One Tb kit–20,000 tests (Cisbio Bioassays, cat. No. 62IPAPEC; Codolet, France);
- cAMP dynamic 2 kit–20,000 tests (Cisbio Bioassays, cat. No. 62AM4PEC; Codolet, France);
- Phospho-ERK1/2 (Cellul’erk)–10,000 tests (Cisbio Bioassays, cat. No. 64ERKPEH; Codolet, France);
- Microtest™ Tissue Culture Plate, 96 Well, Clear (Becton Dickinson, cat. No. 353072; Franklin Lakes, NJ, USA);
- Small volume 384-well plate (white) (Greiner Bio-One, cat. No. GR-784075; Monroe, NC, USA);
- 384 OptiPlate (white) (PerkinElmer, cat. No. 6007290; Waltham, MA, USA);
- TopSeal-A 384, Clear Self-Adhesive Topseal for 384-well Microplates (PerkinElmer, cat. No. 6005250; Waltham, MA, USA).

### 3.2. Reagents

- Hank’s Balanced Salt Solution (HBSS), no Ca^2+^, no Mg^2+^, no phenol red (Invitrogen, cat. No. 14175129; Paisley, UK). Note: preferable with Ca^2+^ and Mg^2+^ when not testing a calcium-sensing receptor;
- Dulbecco’s Phosphate-Buffered Saline (DPBS), no Ca^2+^, no Mg^2+^ (Invitrogen, cat. No. 14190169; Paisley, UK). Note: preferable with Ca^2+^ and Mg^2+^ when not testing a calcium sensing-receptor;
- LiCl (Sigma-Aldrich, cat. No. 310468; St Louis, MO, USA). Note: prepare 1 M in ultra-pure H_2_O
- Cell Dissociation Solution (1×) Non-Enzymatic (Sigma-Aldrich, cat. No. C5914; St. Louis, MO, USA);
- Poly-d-Lysine (Sigma-Aldrich, cat. No. P-0899; St Louis, MO, USA). Note: prepare 4 mg/mL in ultra-pure H_2_O;
- HEPES sodium salt (Sigma-Aldrich, cat. No. H7006; St Louis, MO, USA). Note: prepare 1 M in ultra-pure H_2_O;
- Assay buffer (HBSS + 20 mM HEPES) adjusted to pH 7.4 using either NaOH or HCl. Note: add 1 mM CaCl_2_ and 1 mM MgCl_2_ when testing a receptor that is not activated by these salts. Bovine serum albumin (BSA) can also be added.

### 3.3. Equipment

- EnVision^®^ Xcite Multilabel Plate Reader (PerkinElmer, cat. No. 2104-0020; Waltham, MA, USA) Note: With an HTRF^®^ compatible setup (see Table 2 for an example of a setup).

### 3.4. Procedure

#### 3.4.1. Cell Considerations

For studying the signaling of a GPCR, cells expressing the receptor in question should be used. The cells can either be transiently or stably transfected or having endogenous expression. Cells should be grown using standard conditions.

#### 3.4.2. Ligand Considerations

No special considerations should be taken except interference of high Ca^2+^ concentrations (>10 mM) in the G_q_ pathway assay. Fluorescent compounds should not be a problem because of the time-resolved measurement, but when in doubt, appropriate controls should be performed. Ensuring that measurements of donor and acceptor alone with and with out compound as well as donor signal during assay are not affected would provide assurance. BSA can be added to buffer when testing “sticky” ligands.

#### 3.4.3. **Protocol 1**: Measurement of G_q_ Pathway Activation (d-myo-inositol 1-phosphate, IP_1_)

(1.1) Prepare ligands to be tested in 2× final concentrations in IP_1_ Ligand Buffer (HBSS + 40 mM LiCl) and add 5 μL of ligand solution to each well (in triplicates) in a 384-well OptiPlate. Be sure to dispense at the bottom of the wells. Note: add 1 mM CaCl_2_ and 1 mM MgCl_2_ (final concentrations) to IP_1_ Ligand Buffer when testing a receptor that is not activated by these salts. The concentration of LiCl can be optimized to achieve curves within the linear range of the standard curve. The manufacturer recommends 50 mM.

(1.2) Prepare the cell suspension from a 100 mm tissue culture dish with subconfluent cells as follows: Wash with 5 mL DPBS, add 1–2 mL of Cell Dissociation Solution (37 °C) and incubate the plate at 37 °C and 5% CO_2_ for 5–10 min or until cells are dislodged. Add DPBS to a final volume of 10 mL and pipette up and down until cells are disrupted.

(1.3) Count the cells, spin cell suspension at 900 *g* for approximately 10 min and calculate the volume needed to resuspend the cell pellet in to achieve an optimal cell density. Note: cell density should be optimized. We have found a density of 2 × 10^6^ and 6 × 10^6^ cells/mL for two stably transfected HEK293 cell lines and 1 × 10^7^ cells/mL for a CHO cell line (corresponding to respectively 1 × 10^4^, 3 × 10^4^ and 5 × 10^4^ cells/well) optimal. Here we used 2 × 10^6^ cells/mL.

(1.4) Resuspend the cell pellet in the appropriate volume of 37 °C Assay Buffer and add 5 μL of cell suspension to each well of the 384-well plate and centrifuge plate for 3 s to spin down droplets.

(1.5) Incubate plate for 1 h at 37 °C and then for 15 min at room temperature (RT). Note: the stimulation time at 37 °C can be optimized.

(1.6) During the 15 min incubation prepare the Detection Solution according to the manufacturer’s protocol. Calculate 10 μL per well and 20% in excess. Note: according to the manufacturer the two fluorophores should never be mixed but we have not seen a significant difference between mixing and not mixing. See also (2.7) and Results Section.

(1.7) Add 10 μL of Detection Solution to each well and incubate the sealed plate for minimum 1 h at RT protected from light.

(1.8) Read plate with EnVision^®^ (PerkinElmer, Waltham, MA, USA) with HTRF^®^ compatible setup: excitation at 340 nm and measuring of emitted light at 620 and 665 nm (see Table 2 for an example of a setup).

(1.9) Plot the FRET 665 nm/620 nm ratio, which is inversely proportional to accumulated IP_1_, against the ligand concentrations.

(1.10) For further data analysis make a standard curve using the IP_1_ calibrator as described by the manufacturer, but make sure to make the serial dilution in same buffer constitution and volume as your testing settings. In this protocol we are using a final volume of 20 μL and a 1:1 mixture of Ligand and Assay buffer. Read plate as described in 1.8 and use the standard curve to calculate the amount of produced IP_1_.

(1.11) Make sure the FRET ratio values are within the linear range of the standard curve (Figure 5B) and if not, optimize LiCl concentration, cell amount per well and/or stimulation time.

#### 3.4.4. **Protocol 2**: Measurement of G_s_/G_i_ Pathway Activation (cyclic adenosine 3′,5′-monophosphate, cAMP)

(2.1) Prepare a stock of 10 mM 3-isobutyl-1-methylxanthine (IBMX) in dimethyl sulfoxide (DMSO).

(2.2) From Assay Buffer make cAMP Ligand Buffer by supplementing with IBMX, and if activation of G_i_ is to be tested, forskolin at a suitable concentration. Note: Both IBMX and forskolin concentrations should be optimized. We have used a range of final concentration from 0.1 to 100 μM IBMX and 0.1 to 20 μM forskolin depending on cell line. Remember that the concentration is diluted when cells are being added. Here we used 500 nM IBMX and 5 μM forskolin as final concentrations.

(2.3) Prepare ligands to be tested in 2× final concentration in the prepared cAMP Ligand Buffer.

(2.4) Prepare a cell suspension as described in 1.2 and 1.3 in 37 °C Assay Buffer but at a density optimal for cAMP detection. Note: we have observed densities from 2 × 10^5^ to 2 × 10^6^ cells/mL (corresponding to 1 × 10^3^ to 1 × 10^4^ cells/well) to be optimal depending on cell line. Here we used 4 × 10^5^ cells/mL.

(2.5) Add 5 μL ligand solution and 5 μL cell suspension to a small volume 384-well white plate. Note: make sure the solutions are mixed either by tapping plate or spinning down for 3 s.

(2.6) Incubate plate at RT for 30 min with a lid. Note: the stimulation time should be optimized.

(2.7) Prepare the Detection Solution according to the manufacturer’s protocol. Calculate 10 μL per well and 20% in excess. Note: according to the manufacturer the two fluorophores should never be mixed but we have not seen a significant difference between mixing and not mixing (see Results and Figure 8).

(2.8) Add 10 μL per well of Detection Solution and incubate the plate with lid for minimum 1 h at RT protected from light.

(2.9) Read plate as described in 1.8 and plot the FRET 665 nm/620 nm ratio, which is inverse proportional to accumulated cAMP, against the ligand concentrations.

(2.10) For further data analysis make a standard curve using the cAMP Standard described by the manufacturer. Read with EnVision^®^ as described in 1.8 and use the standard curve to calculate the amount of produced cAMP. Note: we have not observed a difference when using the Diluent (provided in kit) or Assay Buffer to dilute the cAMP Standard. Neither was a difference seen when using cAMP Ligand Buffer with or without IBMX and forskolin (see Results and Figure 8).

(2.11) Make sure all your FRET ratio values are within the linear range of the standard curve (see Figure 6B) and if not, try to optimize IBMX concentration, cell amount per well and/or stimulation time. If G_i_ activation is tested, forskolin concentration in the Ligand Buffer can also be optimized.

#### 3.4.5. **Protocol 3**: Measurement of phosphorylated extracellular signal-regulated kinase 1 and 2 (pERK1/2)

(3.1) The first day, coat a 96-well culture plate (clear) with 50 μL poly-d-Lysine solution (10 mL DPBS + 75 μL poly-d-Lysine 4 mg/mL) and incubate for 30 min.

(3.2) Wash a 100 mm plate containing 80% confluent layer of cells with 5 mL DPBS, add 1 mL of 37 °C 0.05% Trypsin-EDTA and incubate for 1–5 min at 37 °C and 5% CO_2_ until cells are dislodged.

(3.3) Add 9 mL of growth medium to the plate and pipette up and down until cell clumps are disrupted.

(3.4) Count cells and centrifuge cell suspension at 1100 rpm (900 *g*) for 5–10 min.

(3.5) Flick the 96-well culture plate to remove the Poly-d-Lysine solution, wash the plate once with 100 μL DPBS/well and tap it dry on paper towel.

(3.6) Prepare cells by aspirating supernatant and resuspend in an appropriate volume of complete growth medium. Note: we have found a density of 1 × 10^6^ cells/mL for a stable HEK293 cell line and 2 × 10^6^ cells/mL for a stable CHO cell line (corresponding to respectively 5 × 10^4^ and 1 × 10^5^ cells/well) optimal. Here we used 1 × 10^6^ cells/mL.

(3.7) Add 50 μL cell suspension per well to the 96-well plate and incubate overnight at 37 °C and 5% CO_2_.

(3.8) On the following day prepare the ligand solutions (1×) in Assay Buffer.

(3.9) Wash the 96-well plate twice with 50 μL DPBS using a vacuum manifold. Note: add solutions slowly along the sides of the wells and be careful not to tap plate since stress of cells leads to unwanted ERK1/2 phosphorylation [52].

(3.10) Transfer 50 μL of ligand solution slowly along the sides of the wells and incubate for the desired time. Note: stimulation time should be optimized. We have observed maximum response within 15 min.

(3.11) Prepare the Supplemented Lysis Buffer by mixing 3 mL MilliQ water with 1 mL Lysis Buffer 4× (from kit). Then remove 40 μL and add 40 μL of Blocking Reagent 100× (from kit) and mix gently. Note: allow reagents to adapt to RT before mixing.

(3.12) Remove ligand solution from the plate by carefully aspirating with a vacuum manifold. Add 50 μL of the Supplemented Lysis Buffer, add a lid and incubate at RT shaking at 450 rpm for 1 h. Note: the lysis time can be optimized.

(3.13) Prepare the Detection Solution according to the manufacturer’s protocol and add 4 μL to each well in a 384-well small volume white plate. Note: use every second row so transfer from the 96-well format is possible.

(3.14) Transfer 16 μL of cell lysate to the 384-well plate and incubate the plate with lid for 2 h at RT protected from light.

(3.15) Read plate as described in 1.8 and plot the FRET 665 nm/620 nm ratio, which is directly proportional to phosphorylation of ERK1/2, against the ligand concentrations.

(3.16) Further data analysis is not possible since a standard curve cannot be created. It should however be stated that controls are very important since even small mechanical movements can generate a pERK1/2 signal [52]. Use the suggested controls given by the manufacturer. Note: we have observed higher pERK1/2 response for some receptors than the signal achieved with the provided “high-level” control.

#### 3.4.6. Data Analysis

Data analysis was carried out using Prism GraphPad version 5.0a for Mac OS X (GraphPad Software, San Diego, CA, USA). Concentration-response curves were fitted by nonlinear regression using the following equation for sigmoidal concentration-response function with variable slope:

(1) $$R=R_{\text{min}}+\frac{R_{\text{max}}-R_{\text{min}}}{1+10^{\left(\text{log }EC_{50}-X\right)}\cdot n_{\text{H}}}$$

in which *X* is the logarithm of the concentration, *R* is the response, *R*_max_ is the maximal response, *R*_min_ is the minimal response, *EC*_50_ is the concentration giving half-maximum response, and *n*_H_ is the Hill coefficient.

## 4. Conclusions

This technical note paper describes optimized protocols that will allow thorough studying of GPCR signaling in a fast, substantial and robust manner. The time-resolved homogenous format and the use of a highly specific FRET signal provide several advantages compared to other assays. We have applied these protocols for studying the multidimensional signaling properties of the calcium-sensing receptor with great success. In our experience few optimizations are needed to be able to apply this methods for the studies of other GPCRs.

## Figures and Tables

**Table 1.** Overview of assay conditions used for studying CaSR with an HTRF^®^ platform.

Assay	Cell amount per well (plate format)	Stimulation time (min)	Stimulation temp. (°C)	Inhibitor/activator (final concentration)
IP_1_	10^4^ (384; suspension)	60	37	20 mM LiCl
cAMP	2 × 10^4^ (384; suspension)	30	Room temp.	500 nM IBMX + 5 μM forskolin
pERK1/2	5 × 10^4^ (96;adherent, 24 h before)	15	Room temp.	-

**Table 2.** EnVision^®^ Xcite Multilabel Plate Reader setup for HTRF^®^ measurement. CWL: center wavelength; BW: bandwidth.

System	Setting
Excitation	Top
Emission	Top
Second emission	Top
Excitation filter	UV (TRF) 340 (CWL = 340 nm, BW = 60 nm)
Emission filter	APC 665 (CWL = 665 nm, BW = 7.5 nm)
Second emission filter	Europium 615 (CWL = 615 nm, BW = 8.5 nm)
Measurement height	10.2 mm
Cycle (time between flashes)	2000 μs
Delay	150 μs
Number of flashes	100
Number of flashes for 2nd detector	20
Number of sequence windows	1
Total time of windows (measurement time)	300 μs
